# Identification of a solo acylhomoserine lactone synthase from the myxobacterium *Archangium gephyra*

**DOI:** 10.1038/s41598-021-82480-1

**Published:** 2021-02-04

**Authors:** Hanan Albataineh, Maya Duke, Sandeep K. Misra, Joshua S. Sharp, D. Cole Stevens

**Affiliations:** Department of BioMolecular Sciences, University of Mississippi, University, MS USA

**Keywords:** Microbiology, Bacteriology

## Abstract

Considered a key taxon in soil and marine microbial communities, myxobacteria exist as coordinated swarms that utilize a combination of lytic enzymes and specialized metabolites to facilitate predation of microbes. This capacity to produce specialized metabolites and the associated abundance of biosynthetic pathways contained within their genomes have motivated continued drug discovery efforts from myxobacteria. Of all myxobacterial biosynthetic gene clusters deposited in the antiSMASH database, only one putative acylhomoserine lactone (AHL) synthase, *agpI*, was observed, in genome data from *Archangium gephyra*. Without an AHL receptor also apparent in the genome of *A. gephyra*, we sought to determine if AgpI was an uncommon example of an orphaned AHL synthase. Herein we report the bioinformatic assessment of AgpI and discovery of a second AHL synthase from *Vitiosangium* sp. During axenic cultivation conditions, no detectible AHL metabolites were observed in *A. gephyra* extracts. However, heterologous expression of each synthase in *Escherichia coli* provided detectible quantities of 3 AHL signals including 2 known AHLs, C8-AHL and C9-AHL. These results suggest that *A. gephyra* AHL production is dormant during axenic cultivation. The functional, orphaned AHL synthase, AgpI, is unique to *A. gephyra*, and its utility to the predatory myxobacterium remains unknown.

Ubiquitous throughout soils and marine sediments, myxobacteria utilize cooperative features to facilitate uniquely social lifestyles and exhibit organized predation of microbial prey^1–3^. Often attributed to their predatory capabilities, an extraordinary number of biologically active specialized metabolites have been discovered from myxobacteria^4–8^. Interest in this chemical space and the therapeutic potential associated with each elucidated natural product has motivated significant efforts towards continued discovery. Our recent survey of the unexplored, biosynthetic gene clusters from myxobacteria included in the antiSMASH database determined that the potential for such discovery from cultivable myxobacteria remains high^9–12^. An oddity reported by this survey was the presence of a solo acylhomoserine lactone (AHL) synthase within the genome of the myxobacterium *Archangium gephyra*^12–14^. As obligate cooperators numerous signaling systems have been associated with the coordination of myxobacterial motility and predation including A-signal, a quorum-like signal. However, no myxobacteria have been observed to produce AHL quorum signals.

Acylhomoserine lactone quorum signaling (QS) systems are abundant throughout Proteobacteria at-large^15^. Considered autoinducers, AHLs bind to LuxR-type receptors which in turn induce expression of LuxI-type AHL synthases. While a recent assessment of LuxR receptors included within or nearby specialized metabolite biosynthetic gene clusters (BGCs) reported the presence of a putative LuxR receptor from the marine myxobacterium *Haliangium ochraceum* DSM 14,365, no AHL quorum signals or functional LuxI-type AHL synthases have been reported from myxobacteria^16^. Additionally, the presence of putative LuxR receptors within numerous members of the genera *Myxococcus* and *Corallococcus* has been reported in a recent survey of myxobacterial signaling proteins^17^. Intriguingly, the model myxobacterium *Myxococcus xanthus* demonstrates enhanced predatory features when exposed to a variety of exogenous AHLs despite having no obvious LuxR receptor within its genome^18^. This phenomenon, often referred to as “eavesdropping,” has become a generally accepted cornerstone in hypotheses surrounding interspecies cross talk within polymicrobial communities, and the presence of solo or orphan LuxR receptors from species that do not produce AHL signals supports such communication^18–25^. Putative solo-LuxR transcription factors with no accompanying LuxI synthases account for the majority of annotated LuxR proteins. However, as with *M. xanthus*, there are no LuxR receptors apparent in the genome of *A. gephyra*. This suggests that the observed LuxI-type synthase, AgpI, from *A. gephyra* is a solo-LuxI synthase. Considering the abundance of AHL QS systems throughout Proteobacteria other than myxobacteria, the uniqueness of this AHL synthase from *A. gephyra*, and the generalist diet of predatory myxobacteria that includes large swaths of AHL signaling proteobacteria, supports the assumption that *agpI* might have been acquired horizontally^3,26–28^. Conversely, the benefit AHL production might provide a predatory myxobacterium remains non-obvious. Herein we report bioinformatic analysis, functional assessment, and heterologous expression of the myxobacterial AHL synthase AgpI.

## Results

### AgpI is highly homologous to functional AHL synthases

**Figure 1:**
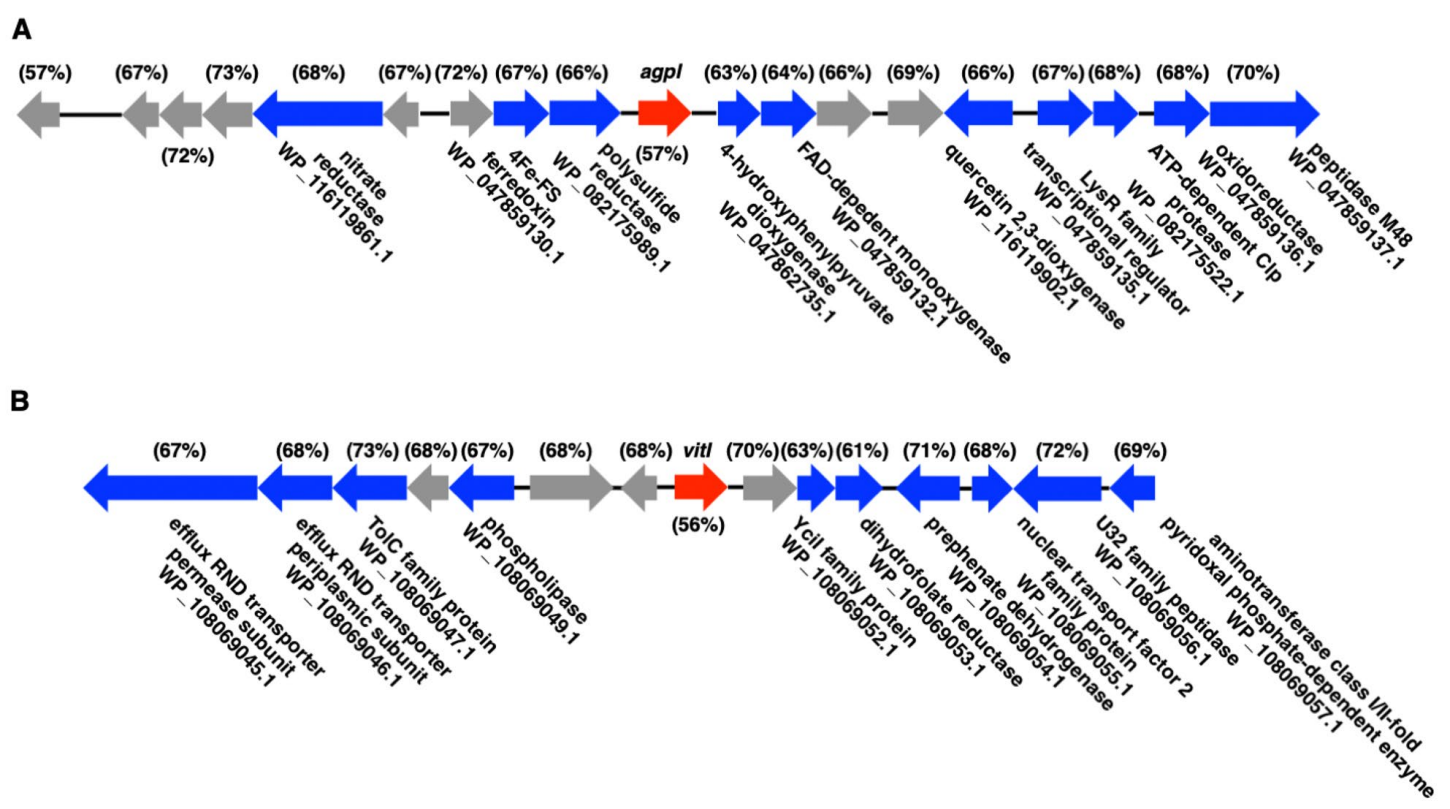
(**A**) Cluster 32 from *A. gephyra* deposited in the antiSMASH database which includes the putative AHL synthase, *agpI*. (**B**) Genomic context for *vitI* from Vitiosangium sp. All annotated features within NCBI are labelled and all hypothetical features are in grey. Percentage GC content for each gene within the cluster provided for comparison and depicted in parentheses.

**Figure 2:**
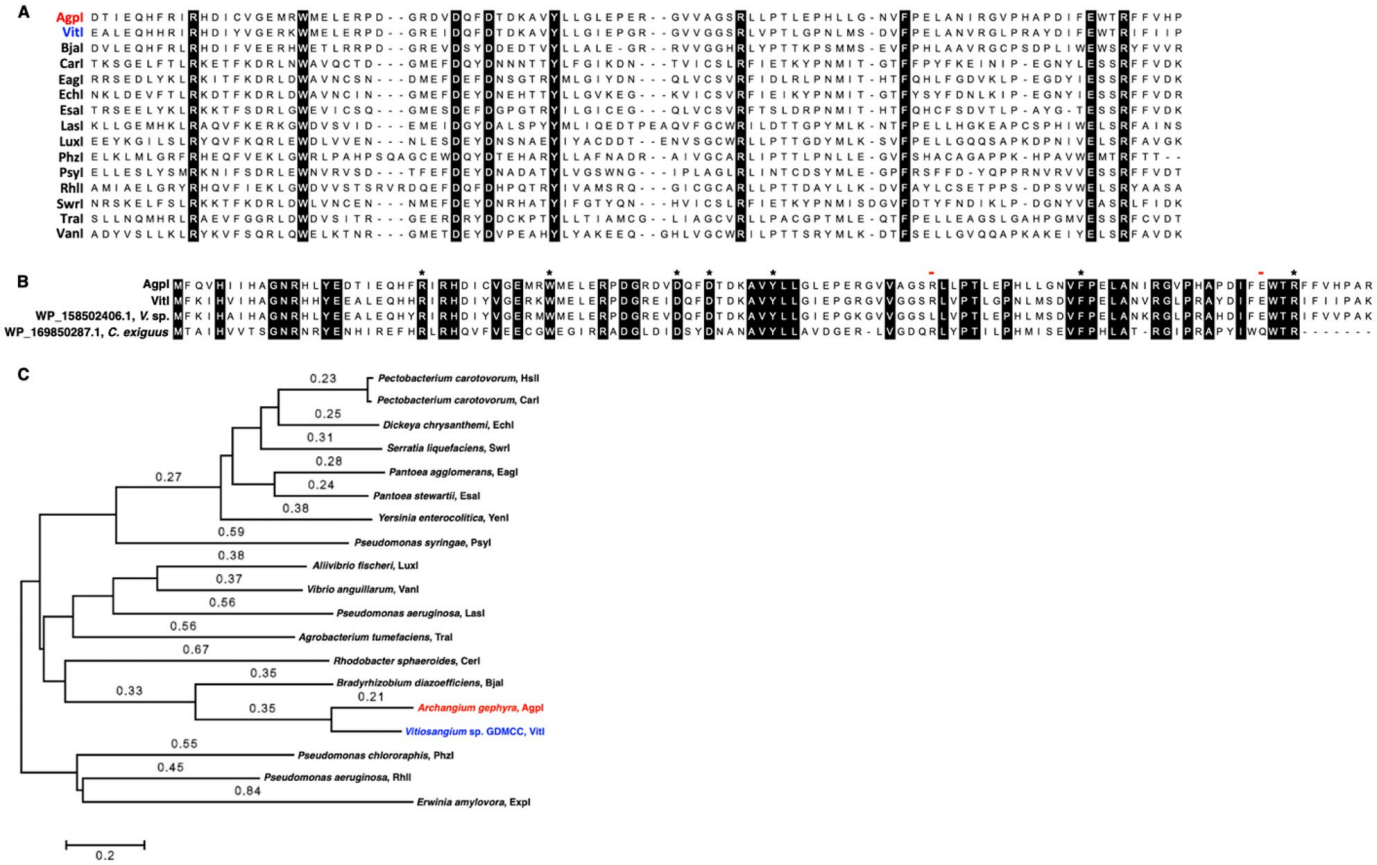
(**A**) Alignment of LuxI synthases including AgpI and VitI with conserved residues boxed in black. (**B**) Alignment of myxobacterial features homologous to AgpI with asterisks (*) indicating conserved residues associated with the LasI autoinducer domain (COG3916) and red lines (-) indicating residues from the LasI domain that are not conserved. (**C**) Minimum Evolution tree including AgpI and VitI rendered in MEGA7 using ClustalW aligned with AHL synthases experimentally confirmed to produce AHLs (68). Branch lengths ≤ 0.2 not depicted.

Located in the 20.6 kb BGC referenced as cluster 32 from *A. gephyra* (NZ_CP011509) in the antiSMASH database (5.1.1) the 210 aa gene product, AgpI (WP_047862734.1), is annotated as a putative autoinducer synthesis protein homologous to the GNAT family *N*-acetyltransferase, LuxI class of AHL synthases (Fig. 1A)^9,10,20^. None of the other annotated features neighboring *agpI* are obviously associated with AHL quorum signaling systems. Assessment of AgpI (WP_047862734.1) with highly homologous LuxI synthases using blastp against the non-redundant protein sequences database provided 2 additional putative AHL synthases within the genome of another myxobacterium, *Vitiosangium* sp. GDMCC 1.1324. These included a GNAT family *N*-acetyltransferase deemed VitI (WP_108069305.1) with 68.12% identity when comparing amino acid sequence data with AgpI (Fig. 1B) and an annotated autoinducer synthase protein (WP_158502406.1) with 69.52% identity with AgpI amino acid sequence^29^. The absence of genome data for *Vitiosangium* sp. in version 4.2.1 of the antiSMASH database explains the omission of this putative AHL synthase from our previous survey of myxobacterial biosynthetic space^12^. The next highest scoring sequence from this analysis, a GNAT family *N*-acetyltransferase (WP_055459978.1) from *Chelatococcus sambhunathii* has 96% coverage and 56.44% identity with AgpI^30,31^. When restricting the blastp search to only provide results from myxobacteria (taxid: 29), only 1 other GNAT *N*-acetyltransferase (WP_169850287.1) from *Corallococcus exiguus* was found to have > 45% identity with AgpI albeit at 50% coverage. Interestingly, an alignment of these 4 putative LuxI-type AHL synthases from myxobacteria revealed that only AgpI and VitI possessed all 8 of the highly conserved residues associated with the LasI autoinducer domain (COG3916) (Fig. 2B). Alignment and phylogenetic analysis of AgpI and VitI against an assortment of 17 LuxI-type synthases experimentally validated to produce AHL QS molecules, suggests common ancestry with the AHL synthases LuxI, LasI, and TraI from *Aliivibrio fischeri*, *Pseudomonas aeruginosa*, and *Rhizobium radiobacter*, respectively (Fig. 2A, C)^32–43^. Utilizing the genomic enzymology web tool EFI-EST developed by the Enzyme Function Initiative (EFI) to construct a sequence similarity network (SSN) that included 1001 LuxI-type AHL synthases (PF00765) as nodes clustered in different groups and 124,346 edges, both AgpI and VitI are included in the central cluster family that contains the vast majority of homologous LuxI-type AHL synthases (Supplemental Figure 1)^44^.

Interestingly, although AgpI and VitI are highly homologous and both include all of the conserved residues associated with LuxI synthases, they share no neighboring similarities when comparing genomic context and surrounding features (Fig. 1). Considering the typical GC-rich genomes of myxobacteria, we sought to compare the GC content of the genes surrounding AgpI and VitI to determine if either were obviously less GC-rich which would support horizontal acquisition. The genomes of both *A. gephyra* and *Vitiosangium* sp are 69.4% and 68.3% GC, respectively. The GC content of AgpI (57%) and VitI (56%) are indeed lower than the genome GC content of each myxobacterium as well as the averages of the genes surrounding them (Fig. 1). A comparison of the genomic context for AgpI (Supplemental Figure 2A) and VitI (Supplemental Figure 2B) as well as the LuxI/GNAT *N*-acetyltransferases with the highest homology from our original blastp analysis of AgpI, including (WP_158502406.1) from *Vitiosangium* sp. (Supplemental Figure 2C), (WP__119746752.1) from *Paracoccus* sp., (WP_055459978.1) from *Chelatococcus sambhunathii*, and (WP_008839764.1) from *Mesorhizobium alhagi* (Supplemental Figure 2D), did not provide an obvious genomic island that might have been included in a hypothetical horizontal transfer event.

Utilizing the BiG-SCAPE platform^45^ which uses a modified iteration of clusterblast, we generated a similarity network from a total of 6627 biosynthetic gene clusters predicted to include LuxI-type AHL synthases from the antiSMASH database^9,10^ including annotated LuxI-type AHL synthases. The biosynthetic gene cluster from *A. gephyra* that includes AgpI appears as a singleton within the resulting similarity network and no pathway-level homology with the other 6627 AHL-containing gene clusters was observed (Supplemental Figure 3). Ultimately these analyses did not provide a clear candidate LuxI-containing gene cluster from which AgpI was acquired; these results do not preclude the possibility that AgpI was indeed acquired as a genetic insertion with various additional features including an associated LuxR-type receptor that have since been lost due to genome reduction. Overall, we suggest that the shared ancestry observed from phylogenetic analysis of AgpI and VitI with known LuxI synthases and highly conserved active site residues suggest both AgpI and VitI are indeed LuxI synthases as originally predicted by antiSMASH.

**Figure 3:**
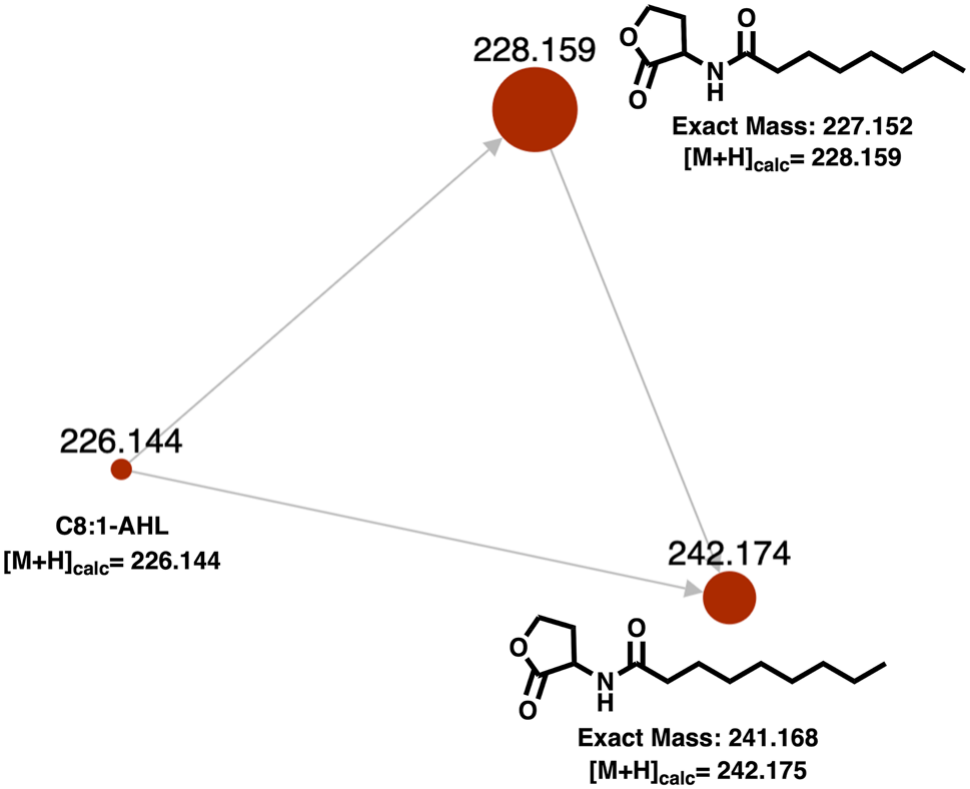
Molecular family from the molecular network of LC–MS/MS datasets from extracts of heterologous *E. coli* expressing AgpI rendered by GNPS (50). Detected m/z values from raw data positioned over each node with node diameter depicting associated intensities for each AHL.

### Absence of a cognate AHL receptor in the genome of *A. gephyra*

While no obvious AHL-binding LuxR homolog was identified in the chromosome of *A. gephyra*, we sought to determine the presence of any potential AHL-binding domain using the conserved sequence for autoinducer binding domains (PF03472). Utilizing the blastp suite at NCBI, we assessed all 3014 domains within the Pfam database classified as autoinducer binding domains for homology against the deposited genome of *A. gephyra*^14,46^. No features within the proteome of *A. gephyra* were sufficiently homologous to be considered to include an autoinducer binding domain. We next queried the Hidden Markov Model (HMM) associated with autoinducer binding domains deposited in Pfam against the proteome of *A. gephyra* using HMMSEARCH^47,48^. The most significant hit (E-value 0.0015) a PAS domain S-box-containing protein also annotated as a GAF-domain-containing protein (WP_053066299.1) does not include significant sequence homology with LuxR-type, AHL receptors.

Utilizing blastp, the genome of *A. gephyra* was also assessed for features homologous to the alternative AHL receptors AinR (AAW85531.1) and LuxN (BAF43687.1)^49,50^, which do not include the conserved autoinducer binding domain associated with LuxR. No homologues with significant homology (> 30% identity) for either alternative receptor were observed. The only resulting features were an annotated response regulator (WP_047859337.1; 43% coverage and 27% identity with AinR) and an annotated MASE1 domain-containing protein (WP_047860847.1; 60% coverage and 20.04% identity with LuxN). Although the annotated MASE1 domain-containing protein is predicted to include an N-terminal transmembrane region, considered to be the recognition site for AHL quorum signals in LuxN, the only homology was associated with the C-terminal response regulatory receiver domain (PF00072.24) of LuxN.

Similar analysis of *Vitiosangium* sp. GDMCC 1.1324 provided a highly homologous LuxR-type receptor (WP_108076247.1). While the AHL receptor identified in the genome of *Vitiosangium* sp. is not clustered near *vitI* as is typical of LuxI-LuxR type synthase-receptor pairs, we cannot assume both are unpaired orphans and instead consider VitI might not be a truly solo AHL synthase (Supplemental Figure 4). Interestingly, the solo LuxR from *Escherichia coli* SdiA (PRK10188)^51^ was the highest scoring domain hit provided by blastp analysis of the LuxR from *Vitiosangium* sp. From these data we determined AgpI to be an orphaned AHL synthase without any cognate LuxR, AinR, or LuxN receptor present in the genome of *A. gephyra*, and despite the unclustered nature of the LuxR homologue identified in the genome of *Vitiosangium* sp., VitI and the annotated autoinducer synthase (WP_158502406.1) cannot be considered solo LuxI synthases without further investigation*.*

**Figure 4:**
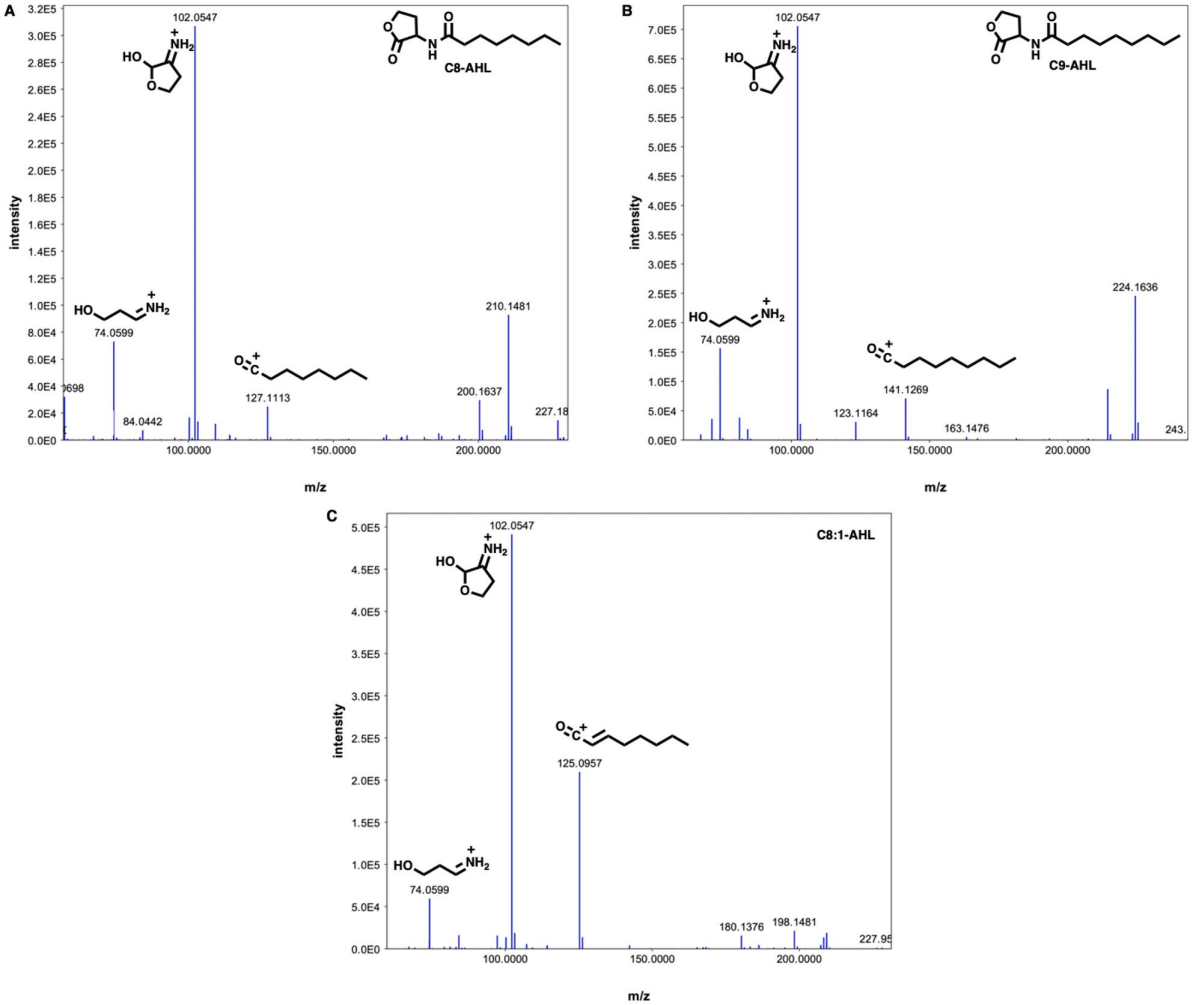
MS/MS fragmentation spectra with diagnostic fragments indicated for each AHL detected in extracts from heterologous *E. coli* expressing AgpI.

### *A. gephyra* does not produce AHLs during axenic cultivation

Cultivation of *A. gephyra* on VY/2 agar plates at 30 °C for 21 days provided fully developed, wispy myxobacterial swarms encompassing the entirety of the plate surface. Homogenized agar and cellular contents were extracted using traditional organic phase techniques to provide extracts for LC–MS/MS analysis. The resulting datasets from LC–MS/MS analysis of *A. gephyra* extracts were analyzed against datasets generated from analytical standards for a variety of AHLs including C9-AHL, C8-AHL, and C11-AHL to determine the presence of any produced AHL-like metabolites. Data from resulting mass spectra were scrutinized using the Global Natural Products Social Molecular Networking (GNPS) platform to generate molecular networks depicting similarities in detected metabolite scaffolds inferred from ionized fragment commonalities^52^. No metabolites that included the diagnostic AHL fragments at 102.0547 m/z and 74.0599 m/z associated with the core homoserine lactone moiety were detected in extracts from *A. gephyra*^53,54^. This data supports any one of the following conclusions *A. gephyra* does not produce AHL-like metabolites when grown axenically but may be active under other growth conditions; metabolites produced by AgpI do not possess structural similarity with typical AHL metabolites; or AgpI is simply nonfunctional. Silent or dormant BGCs are commonly observed during natural product discovery efforts, and various strategies to activate silent BGCs have been developed including addition of exogenous chemical elicitors and heterologous expression of silent BGCs in an alternative host^55,56^.

### Exogenous AHLs do not activate AgpI

Considering the typical autoinduction of LuxI synthases, we sought to determine if exogenous AHL metabolites might induce AgpI and provide observable AHL-like metabolites from *A. gephyra*. These experiments were conducted despite the absence of a feature that includes the conserved autoinducer domain encoded in the genome of *A. gephyra* in an effort to determine if a regulatory element with a non-typical AHL binding domain might induce expression of AgpI. Experiments introducing the deuterated AHLs *N*-hexanoyl-L-homoserine lactone-*d*3 (C6-AHL-*d*3) and *N*-butyryl-L-homoserine lactone-*d*5 (C4-AHL-*d*5) to *A. gephyra* plates at 30 μM after two weeks of growth at 30 °C were conducted to determine if exogenous AHLs induce AgpI activity. Deuterium-labelled analogs of C6-AHL and C4-AHL were utilized to provide the ability to decouple exogenous signals from structurally similar AHLs potentially induced by exogenous AHL introduction. Using LC–MS/MS and molecular networking as previously described, no metabolites possessing the core homoserine lactone moiety were detected in the deuterated AHL-exposed extracts from *A. gephyra* suggesting that AgpI activity is not induced by exogenous AHLs. Ultimately, these experiments confirm that the exogenous AHL signals C6-AHL and C4-AHL do not induce biosynthesis of AHL metabolites from *A. gephyra*.

### Heterologous expression of AgpI confirms functional production of AHLs

To explore the functionality of both AgpI and VitI and assumed biosynthesis of AHL-like metabolites, IPTG-inducible codon-optimized constructs of *agpI* and *vitI* included in replicating pET-28b( +) plasmids suitable for expression in *Escherichia coli* BL21 were purchased. Heterologous expression of AgpI and VitI, subsequent extraction, LC–MS/MS analysis, and evaluation of molecular networks rendered by GNPS as previously described, provided a cluster family including 2 of 3 total nodes identified as C8-AHL (228.159 m/z) and C9-AHL (242.174 m/z) from internal GNPS public datasets as well as a third AHL metabolite detected at 226.144 m/z (Fig. 3)^52^. This cluster family was identical in both heterologous expression experiments suggesting that AgpI and VitI produce the same 3 AHL metabolites when heterologously expressed in *E. coli* with similar detected intensities for each AHL. Both C8-AHL and C9-AHL were confirmed to be present in AgpI and VitI extracts using analytical standards. Based on associated intensities, C8-AHL was the most abundant and the metabolite detected at 226.144 m/z was the least abundant AHL. No AHL-like entities were detected in control extracts from *E. coli* containing no plasmid and *E. coli* containing an empty pET28b expression plasmid (Supplemental Figures 5 and 6). From the mass difference between C8-AHL and the unknown AHL detected at 226.144 m/z (2.015 Da measured vs. 2.01565 theoretical), as well as shared fragmentation patterns, we suggest the metabolite detected at 226.144 m/z to likely be an unsaturated analog of C8-AHL (Fig. 4). From these experiments we determined that both AgpI and VitI are functional AHL synthases capable of producing the previously characterized AHLs C8-AHL and C9-AHL. These results suggest *A. gephyra* could produce AHLs and likely requires environmental cues or specific nutrients not present during our axenic cultivation conditions.

## Discussion

Ultimately we conclude that the myxobacteria *A. gephyra* and *Vitiosangium* sp. encode functional AHL synthases that produce the AHL signals C8-AHL and C9-AHL when heterologously expressed in *E. coli*. Considering the strong precedent for heterologous expression of AHL synthases in *E. coli* to determine produced AHL metabolites, this suggests that both *A. gephyra* and *Vitiosangium* sp. are capable of producing one or all of the observed AHL signals and that AgpI is merely silent or cryptic during axenic cultivation of *A. gephyra*^55,57–61^. However, provided the subtle differences in LuxI synthase homologies and resulting chemical diversity of produced AHLs^34^, we should also consider that these synthases could instead utilize an acyl-ACP or CoA thioester precursor not available to the heterologous *E. coli* host, and we are actively exploring cultivation conditions that might induce native AHL production from *A. gephyra*^55,59^. While numerous bacteria have been observed to possess orphaned LuxR-type AHL receptors, a functional solo LuxI synthase without any cognate LuxR receptor also present in the genome has yet to be reported^21,23,24,62^. Although a functional orphaned LuxI-type synthase capable of producing AHLs has been reported from the sponge symbiont *Ruegeria* sp. KLH11, the strain also harbors 2 pairs of clustered LuxI/LuxR homologues^23,63^. We suggest that production of AHL quorum signals by myxobacteria would support the theoretical benefits of interspecies cross talk similar to functional, solo LuxR receptors^24,64–66^. We also propose that the more typical abundance of orphan LuxR receptors compared to the seemingly exceptional solo LuxI synthase reported here might correlate with the rarity of cooperative generalist predators^21,28^. The absence of any AHL metabolites during axenic cultivation of *A. gephyra* suggests an unknown regulatory mechanism independent from a LuxR receptor might be involved. However, previously reported eavesdropping by *M. xanthus* and response to exogenous AHLs despite the absence of any AHL receptor with homology to LuxR suggests myxobacteria may possess an undiscovered, alternative means of AHL detection and response^18^. An alternative explanation for the absence of AHL metabolites in the extracts of *A. gephyra* would be that AgpI is merely a non-functional feature that has been acquired but is not utilized. Although this seems unlikely due to the presence of additional LuxI-type synthases in the genome of *Vitiosangium* sp. and the understanding that specialized metabolite biosynthetic genes are often silent or unexpressed during axenic cultivation^12,55,56^, this explanation should be considered until either myxobacterium is observed to produce AHLs metabolites. While the benefit afforded predatory myxobacteria remains unclear, production of AHL signals known to regulate QS-associated physiological functions such as biofilm formation, specialized metabolism, and motility offers some insight^15^. Predatory disruption of any one of these functions would likely benefit the fitness of *A. gephyra* by improving predation of quorum signaling prey. For example, *Pseudomonas putida* biofilm formation is negatively regulated by the presence of AHLs^67,68^, and biofilm formation is commonly associated with predator avoidance^69,70^. Myxobacterial production of AHLs would therefore inhibit biofilm formation of *P. putida* which would benefit predation. However, without direct evidence of myxobacterial AHL production any interplay between AHL biosynthesis and predator–prey interactions remains hypothetical. Overall, we consider the presence of 2 functional AHL synthases within the genomes of 2 predatory myxobacteria provides a unique perspective and supports the continued investigation of small molecule interactions that contribute to microbial community structures.

## Materials and methods

### Cultivation of *A. gephyra*

*Archangium gephyra* (DSM 2261) was initially obtained from German Collection of Microorganisms in Braunschweig was grown at 30 °C on VY/2 agar (5 g/L baker’s yeast, 1.36 g/L CaCl_2_, 0.5 mg/L vitamin B_12_, 15 g/L agar, pH 7.2).

### Bioinformatic assessment of AgpI and VitI

The amino acid sequence for AgpI (WP_047862734.1) was submitted for blastp analysis against the non-redundant protein sequences database. The amino acid sequences for AgpI (WP_047862734.1) and VitI (WP_108069305.1) were submitted to EFI-EST analysis (https://efi.igb.illinois.edu/efi-est/) to construct a sequence similarity network (SSN) of LuxI-type AHL synthases (PF00765) using the default settings. Results from EFI-EST analysis were visualized using Cytoscape (3.8.2) and are provided as supplemental data. Genomic contexts of AgpI (WP_047862734.1), VitI (WP_108069305.1), and the most homologous GNAT family N-acetyltransferases/autoinducer synthesis proteins according to blastp: including (WP_158502406.1) from *Vitiosangium* sp., (WP__119746752.1) from *Paracoccus* sp., (WP_055459978.1) from *Chelatococcus sambhunathii*, and from *Mesorhizobium alhagi* were analyzed to calculate GC% of each acetyltransferases/autoinducer synthase and the surrounding genes (Fig. 1 and Supplemental Figure 2). Alignments from ClustalW, minimum evolution, and maximum likelihood phylogenetic trees were rendered using either MEGA 7 or MEGA X^71–74^. BiG-SCAPE (https://git.wageningenur.nl/medema-group/BiG-SCAPE) was was utilized locally to generate a sequence similarity network of all BGCs containing putative LuxI AHL synthases downloaded from the antiSMASH database (v2) . The resulting network (Supplemental Figure 3) included a total 6627 nodes and 195,239 edges and was generated using the default parameters in BiG-SCAPE.

### Autoinducer binding site search

All 3014 domains annotated as autoinducer binding domains (PF03472) deposited in Pfam were subjected to blastp analysis against the *A. gephyra* genome (NZ_CP011509.1). For HMMSEARCH analysis, the raw HHM for autoinducer binding domains was downloaded from Pfam (PF03472) and utilized as input for profile-HMM vs protein sequence database via HMMSEARCH with the taxonomy restrictions set to limit analysis to *A. gephyra* or *Vitiosangium* sp. The amino acid sequence for AinR (AAW85531.1) and LuxN (BAF43687.1) was submitted for blastp analysis against the *A. gephyra* (NZ_CP011509) and *Vitiosangium* sp. (NZ_PZOX00000000.1) genomes.

### Heterologous expression of AgpI and VitI in *E. coli*

Constructs of AgpI and VitI codon optimized for expression in *E. coli* situated in pET28b were purchased from Genscript (Piscataway, NJ). Sequence data for these constructs are provided as supplemental data. The heterologous host *E. coli* K207-3 was transformed with each plasmid individually by electroporation (BTX Gemini Sc2, Harvard apparatus) to provide an *E. coli* strain capable of expressing AgpI and an *E. coli* strain capable of expressing VitI. In addition, an *E. coli* negative control was generated by transforming *E. coli* K207-3 with an empty pET28b vector (no AHL synthase construct). The transformed *E. coli* strains were grown at 37 °C in LB broth supplemented with 50 μg/mL kanamycin, induced with IPTG (final concentration of 1.0 mM) at OD_600_ = 0.6, and grown overnight at 14 °C with shaking to facilitate heterologous protein expression. *E. coli* K207-3 with no included plasmid was also included as a negative control and was grown without the addition of kanamycin but otherwise under the same conditions.

### Metabolite extraction and analysis

After 21 days of cultivation, *A. gephyra* plates were manually diced and extracted with excess EtOAc. Pooled EtOAc was filtered and dried *in vacuo* to provide crude extracts for LC–MS/MS analysis. Extracts from controls and heterologous strains of *E. coli* were generated by Amberlite XAD-16 absorber resin (Alfa Aesar) facilitated extraction of clarified culture broths following cell lysis. Resins were removed by filtration and were eluted with MeOH to provide extracts for LC–MS/MS analysis. Extraction for all strains were performed in triplicate.

LC–MS/MS analysis of the extracted samples was performed on an Orbitrap Fusion instrument (Thermo Scientific, San Jose, CA) controlled with Xcalibur version 2.0.7 and coupled to a Dionex Ultimate 3000 nanoUHPLC system. Samples were loaded onto a PepMap 100 C18 column (0.3 mm × 150 mm, 2 μm, Thermo Fisher Scientific). Separation of the samples was performed using mobile phase A (0.1% formic acid in water) and mobile phase B (0.1% formic acid in acetonitrile) at a rate of 6 μL/min. The samples were eluted with a gradient consisting of 5 to 60% solvent B over 15 min, ramped to 95% B over 2 min, held for 3 min, and then returned to 5% B over 3 min and held for 8 min. All data were acquired in positive ion mode. Collision-induced dissociation (CID) was used to fragment molecules, with an isolation width of 3 m/z units. The spray voltage was set to 3600 V, and the temperature of the heated capillary was set to 300 °C. In CID mode, full MS scans were acquired from m/z 150 to 1200 followed by eight subsequent MS2 scans on the top eight most abundant peaks. The orbitrap resolution for both the MS1 and MS2 scans was 120,000. The expected mass accuracy was < 3 ppm. All extracts from heterologous expression experiments for AgpI and VitI performed in triplicate were analyzed with AHL production confirmed in each as described. Extracted-ion chromatographs depicting C8- and C9-AHL detection from *E. coli* expressing AgpI and *E. coli* expressing VitI and absence of detectible quantities of either AHL in extracts from negative controls included as Supplemental Figures 5 and 6.

### Exogenous AHL exposure experiments

Stock solutions (10 mM) of *N*-hexanoyl-L-homoserine lactone-*d*3 (C6-AHL-*d*3) and *N*-butyryl-L-homoserine lactone-*d*5 (C4-AHL-*d*5) (Cayman Chemical) were prepared in DMSO. The required volumes of these stock solutions were filter sterilized and added to 14 days growing *A. gephyra* plates to give a final concentration of 30 μM. After 7 days of exogenous AHL exposure, *A. gephyra* plates were manually diced, extracted with excess EtOAc, and submitted to LC–MS/MS analysis as previously described.

### GNPS dataset

Generated data were converted to .mzXML files using MS-Convert and mass spectrometry molecular networks were generated using the GNPS platform (http://gnps.ucsd.edu)^52^. LC–MS/MS data for this analysis were also deposited in the MassIVE Public GNPS data set (MSV000084574).

### Supplementary material

Sequence data for codon-optimized AgpI and VitI constructs and Supplemental Figures 1–6 are included as supplementary material.

## Supplementary Information

Supplementary Information.
