# Supplementary material for: Identification of a solo acylhomoserine lactone synthase from the myxobacterium Archangium gephyra
Source: Sci Rep. 2021 Feb 4;11:3018. doi: 10.1038/s41598-021-82480-1 (PMC7862692; doi:10.1038/s41598-021-82480-1)

**Identification of a solo acylhomoserine lactone synthase from the myxobacterium *Archangium gephyra***

Hanan Albataineh^1^, Maya Duke^1^, Sandeep K. Misra^1^, Joshua S. Sharp^1^, and D. Cole Stevens^1,#^

Department of BioMolecular Sciences, University of Mississippi, University, MS, USA^a^

**Supplemental Information**

Sequence data for codon-optimized AgpI construct purchased from Genscript:

CATATGTTCCAGGTGCACATCATTCACGCGGGTAACCGTCACCTGTACGAGGACACCATCGAACAACACTTTCGTATCCGTCACGATATTTGCGTTGGCGAGATGCGTTGGATGGAGCTGGAACGTCCGGACGGCCGTGACGTGGATCAGTTCGACACCGATAAGGCGGTTTATCTGCTGGGCCTGGAACCGGAACGTGGTGTGGTTGCGGGTAGCCGTCTGCTGCCGACCCTGGAGCCGCACCTGCTGGGTAACGTGTTCCCGGAACTGGCGAACATCCGTGGCGTTCCGCATGCGCCGGATATTTTTGAGTGGACCCGTTTCTTTGTGCACCCGGCGCGTCGTGAAAGCAACCGTCCGAGCAAGGCGGCGGGTATCATTCAGTGCGCGATCGTGGAGTTCTGCCTGAGCCAAAACATCCGTAAAATTAGCGGCGTTTGCGAAGCGCATTGGCTGCCGCGTCTGCAAGCGCTGGGTTGGAACCCGTGCCTGCTGGGTGAACCGATGCGTAAAGACAGCATGACCATTGTTGGTCTGAGCACCGATATGACCACCGAGGCGCTGGAACGTACCCGTAGCGCGTACGGTATCATTGGCAGCGTGATCGCGCCGGTTAGCATTCCGTTTAACCGTGAATTC

Sequence data for codon-optimized VitI construct purchased from Genscript:

CATATGTTCAAGATCCACGTGATTCACGCGGGTAACCGTCACCACTACGAGGAAGCGCTGGAGCAGCACCACCGTATCCGTCACGATATTTATGTTGGTGAGCGTAAGTGGATGGAGCTGGAACGTCCGGACGGCCGTGAGATCGATCAATTCGACACCGATAAAGCGGTGTACCTGCTGGGCATTGAGCCGGGTCGTGGCGTGGTTGGTGGCAGCCGTCTGGTTCCGACCCTGGGTCCGAACCTGATGAGCGACGTGTTTCCGGAACTGGCGAACGTTCGTGGCCTGCCGCGTGCGTATGATATCTTCGAGTGGACCCGTATTTTTATCATTCCGGCGAAACGTGAAAGCGGTCGTCTGAGCCAGGCGGCGGGTATCGTGTACTGCGGCATTCTGGAATTTTGCCTGAGCCAGCACATCCGTCAACTGAGCGTGGTTTGCGAGGACTATTGGATTCCGCGTCTGCAAGCGCTGGGTTGGAGCCCGGTGCGTCTGGGTGAAGCGATCCTGAAGGACGATATGAGCATCGTTGGCATTACCTGCGATATGACCGAGGAAGCGCTGGCGAAAACCCGTAGCACCTACAGCATCGACGATAGCGTGATGGCGCCGGAGTTCGTTCCGAACGAACTGCTGATGCTGGACTTTAACAGCTGCCAGGAATTC


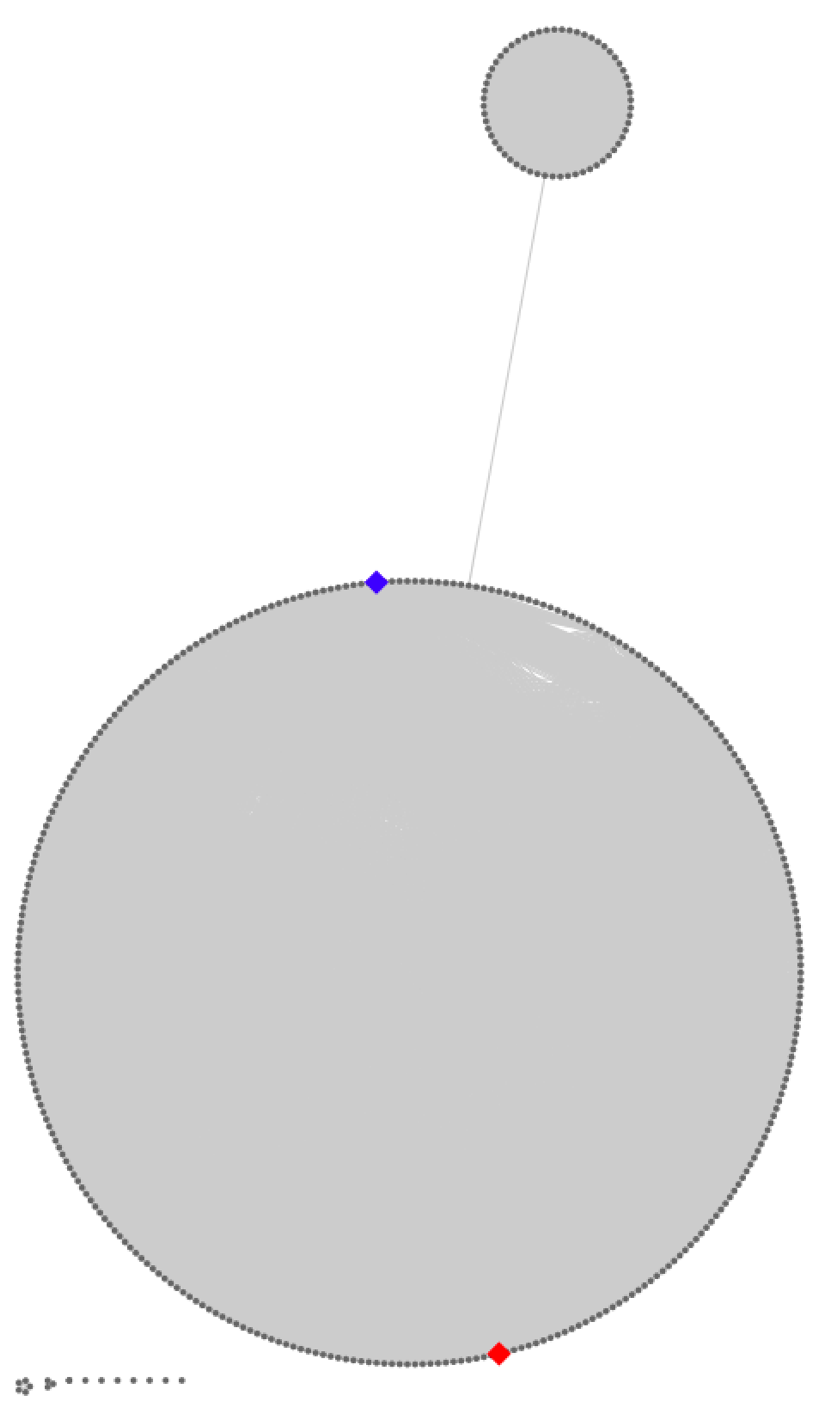


**Supplemental Figure 1:** Sequence similarity network rendered by EFI-EST analysis of AgpI amino acid sequence data with AgpI (red diamond) and VitI (blue diamond) indicated. LuxI-type AHL synthases are represented as grey nodes (a total of 1,001 nodes clustered in 2 groups) with grey edged connect the nodes (a total of 124,346 edges). To reduce complexity all nodes with ≥90% sequence similarity are represented as an individual aggregate node. For Cytoscape visualization, Attribute Circle Layout was applied.


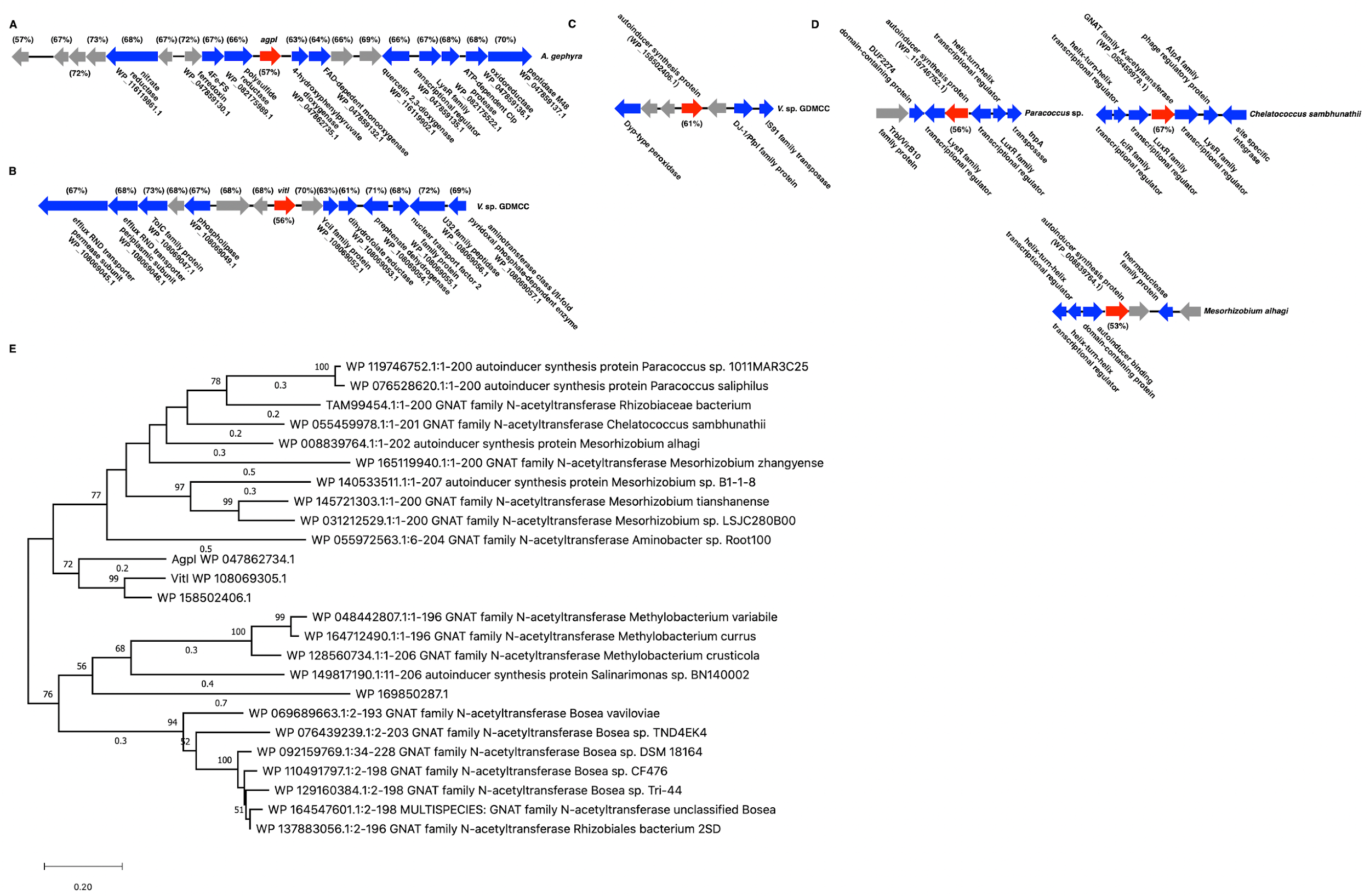

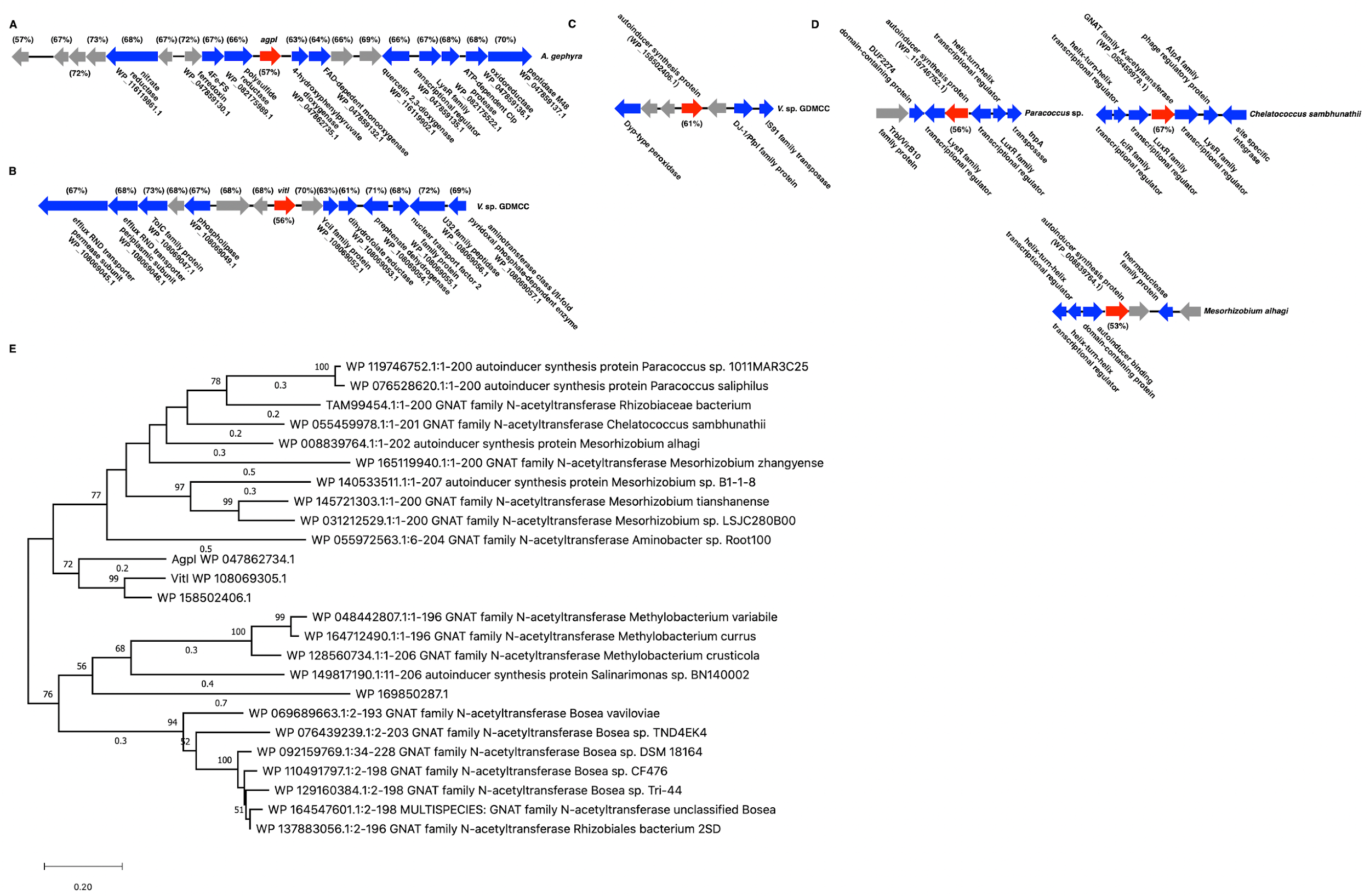


**Supplemental Figure 2:** Genomic context for A) *agpI* (as depicted in Figure 1 from text), B) *vitI* (also as depicted in Figure 1 from text), C) and the resulting GNAT family N-acetyltransferases/autoinducer synthesis proteins with the highest homology as determined by blastp analysis of AgpI as described including (WP_158502406.1) from *Vitiosangium*sp. GDMCC, D) (WP__119746752.1) from *Paracoccus* sp., (WP_055459978.1) from *Chelatococcus sambhunathii*, and (WP_008839764.1) from *Mesorhizobium alhagi* All annotated features within NCBI are labelled and all hypothetical features are in grey. Percentage GC content for each gene depicted in parentheses. E) Phylogenetic tree of homologous features resulting from blastp analysis of AgpI (25 total amino acid sequences) generated using the Maximum Likelihood method and JTT matrix-based model. Percentage of trees in which associated features clustered together is shown above branches with values below 50% not included. Branch lengths < 0.2 not included.

**Supplemental Figure 3:** Sequence similarity network of all biosynthetic gene clusters containing putative LuxI AHL synthases downloaded from the antiSMASH database (v2) with the singleton including AgpI circled. Network includes a total 6,627 nodes and 195,239 edges and was generated using the default parameters in BiG-SCAPE.
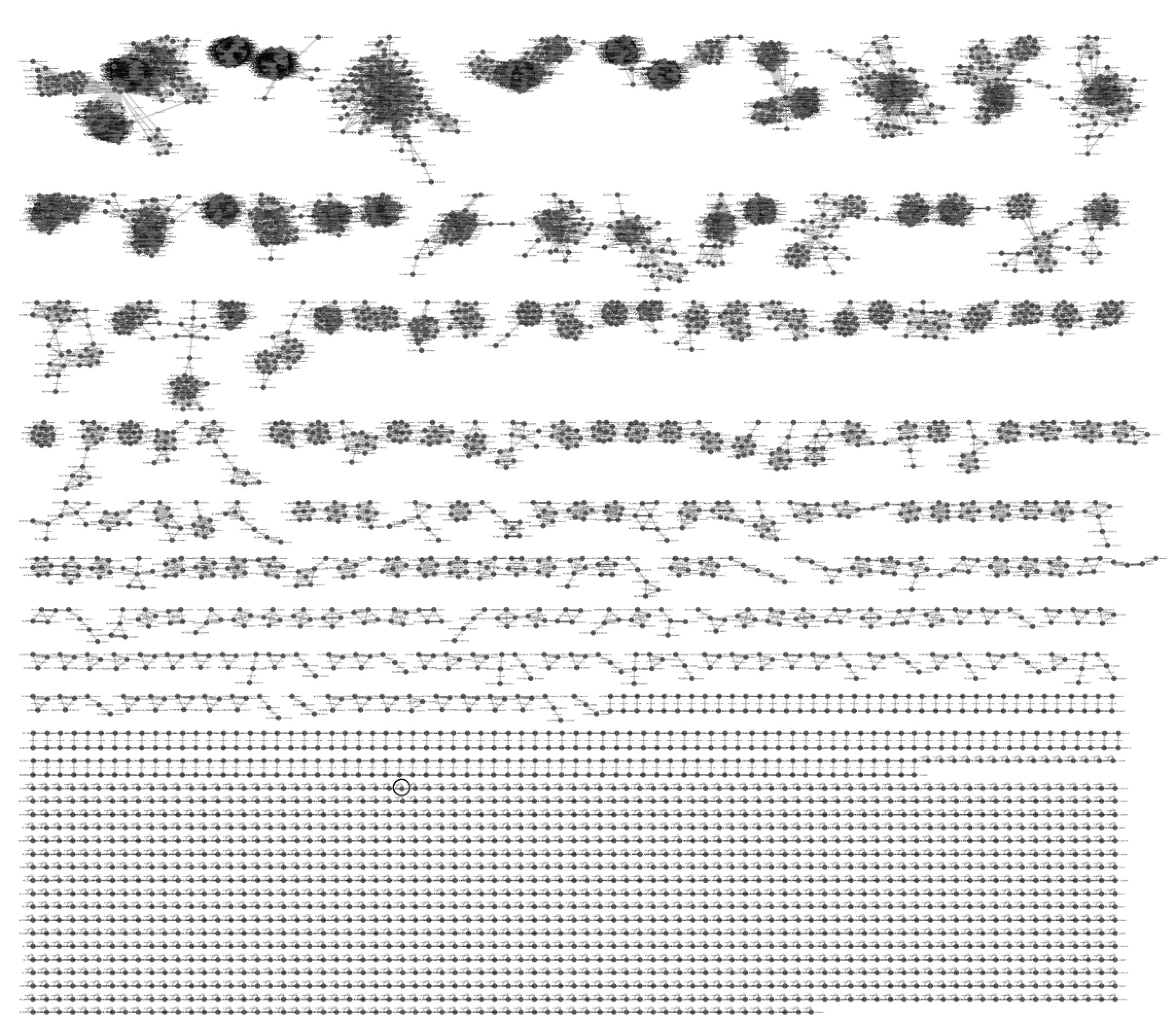


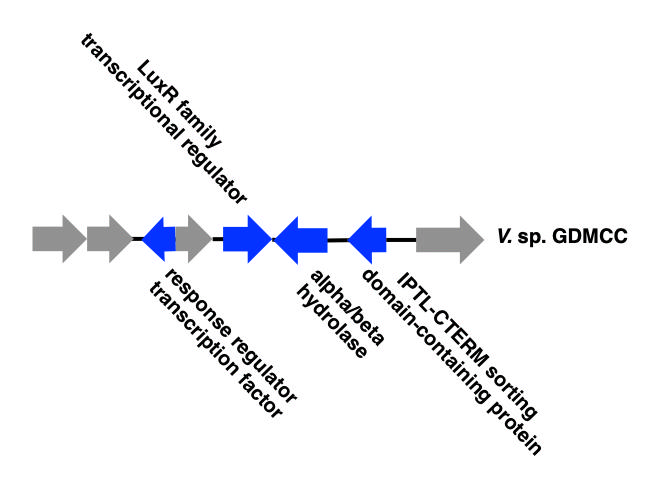


**Supplemental Figure 4:** Genomic region of *V.* sp. GDMCC 1.1324 including the putative LuxR-type receptor (WP_108076247.1) and surrounding genes. All annotated features within NCBI are labelled and all hypothetical features are in grey.

**Supplemental Figure 5:** Extracted-ion chromatograph (228-229 m/z) depicting presence of C8-AHL in extracts from *E. coli* K207-3 heterologously expressing AgpI (blue) and VitI (red) and absence of C8-AHL in extracts from *E. coli* K207-3 + pET28b (green) and *E. coli* K207-3 with no plasmid (pink).
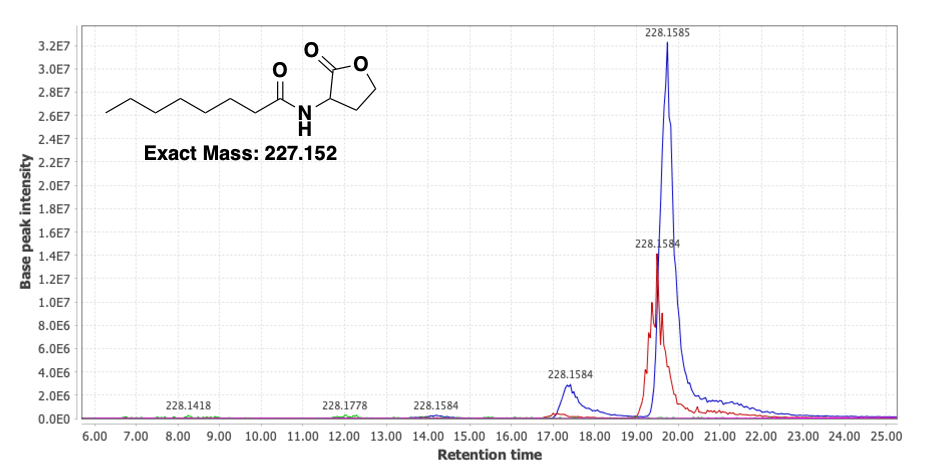


**Supplemental Figure 6:** Extracted-ion chromatograph (242.1-242.2 m/z) depicting presence of C9-AHL in extracts from *E. coli* K207-3 heterologously expressing AgpI (blue) and VitI (red) and absence of C9-AHL in extracts from *E. coli* K207-3 + pET28b (green) and *E. coli* K207-3 with no plasmid (pink).
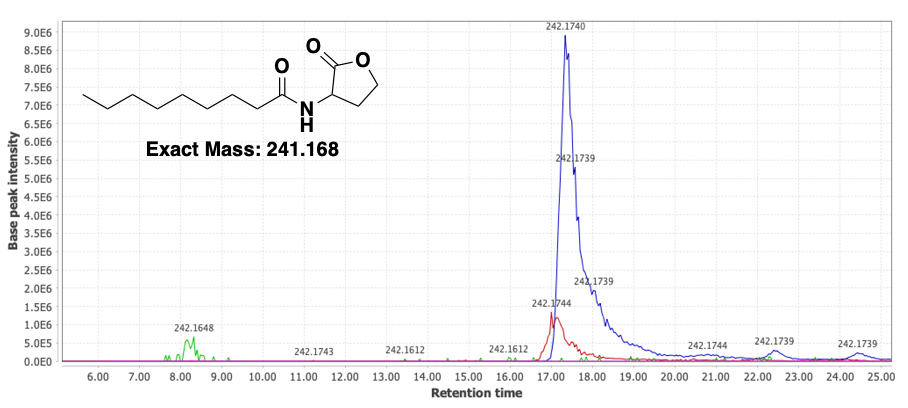

Supplement: Supplementary file 1 — Supplementary Information. [file 41598_2021_82480_MOESM1_ESM.docx]
